# A New Look at the Genus *Solobacterium*: A Retrospective Analysis of Twenty-Seven Cases of Infection Involving *S. moorei* and a Review of Sequence Databases and the Literature

**DOI:** 10.3390/microorganisms9061229

**Published:** 2021-06-05

**Authors:** Corentine Alauzet, Fabien Aujoulat, Alain Lozniewski, Safa Ben Brahim, Chloé Domenjod, Cécilia Enault, Jean-Philippe Lavigne, Hélène Marchandin

**Affiliations:** 1 Laboratoire SIMPA Stress Immunité Pathogènes EA 7300, Université de Lorraine, & Service de Microbiologie, CHRU de Nancy, 54500 Vandœuvre-lès-Nancy, France; c.alauzet@chru-nancy.fr (C.A.); alain.lozniewski@univ-lorraine.fr (A.L.); 2 HydroSciences Montpellier, CNRS, IRD, Université de Montpellier, 34093 Montpellier, France; fabien.aujoulat@umontpellier.fr; 3 Service de Microbiologie, CHRU de Nancy, 54500 Vandœuvre-lès-Nancy, France; s.benbrahim@chru-nancy.fr; 4 Service de Microbiologie et Hygiène Hospitalière, CHU de Nîmes, 30029 Nîmes, France; chloe.domenjod@gmail.com (C.D.); cecilia.enault@chu-nimes.fr (C.E.); 5 VBIC, INSERM U1047, Université de Montpellier, Service de Microbiologie et Hygiène Hospitalière, CHU Nîmes, 30029 Nîmes, France; jean.philippe.LAVIGNE@chu-nimes.fr; 6 HydroSciences Montpellier, CNRS, IRD, Université de Montpellier, Service de Microbiologie et Hygiène Hospitalière, CHU de Nîmes, 30029 Nîmes, France

**Keywords:** *Solobacterium moorei*, human infections, human microbiota, identification, antimicrobial susceptibility, sequence databases, metagenomes, habitat

## Abstract

*Solobacterium moorei* is an anaerobic Gram-positive bacillus present within the oral and the intestinal microbiota that has rarely been described in human infections. Besides its role in halitosis and oral infections, *S. moorei* is considered to be an opportunistic pathogen causing mainly bloodstream and surgical wound infections. We performed a retrospective study of 27 cases of infections involving *S. moorei* in two French university hospitals between 2006 and 2021 with the aim of increasing our knowledge of this unrecognized opportunistic pathogen. We also reviewed all the data available in the literature and in genetic and metagenomic sequence databases. In addition to previously reported infections, *S. moorei* had been isolated from various sites and involved in intra-abdominal, osteoarticular, and cerebral infections more rarely or not previously reported. Although mostly involved in polymicrobial infections, in seven cases, it was the only pathogen recovered. Not included in all mass spectrometry databases, its identification can require 16S rRNA gene sequencing. High susceptibility to antibiotics (apart from rifampicin, moxifloxacin, and clindamycin; 91.3%, 11.8%, and 4.3% of resistant strains, respectively) has been noted. Our global search strategy revealed *S. moorei* to be human-associated, widely distributed in the human microbiota, including the vaginal and skin microbiota, which may be other sources for infection in addition to the oral and gut microbiota.

## 1. Introduction

The genus *Solobacterium* was created in 2000 to classify anaerobic, non-sporulated Gram-positive bacilli isolated from human feces that were phylogenetically distant from the genera *Eubacterium*, *Holdemania*, and *Erysipelothrix*. The nomenclature of the genus referred to a lone bacterial species (*solus* for sole, *bakterion* for small rod). Nowadays, the genus is classified in the family *Erysipelotrichaceae* within the phylum *Firmicutes* and, 20 years after its description, still comprises a unique validated species, *Solobacterium moorei* [1], for which the name *Bulleidia moorei* had also been proposed but not validated.

Three years after its characterization, *S. moorei* was found to be part of the tongue microbiota species and phylotypes that were significantly associated with halitosis [2], a finding that was later confirmed by Haraszthy et al. [3,4]. The fact that the presence of *S. moorei* was found to be significantly correlated with organoleptic scores of halitosis, volatile sulfur compound (hydrogen sulfide) levels, and beta-galactosidase activities was further consistent with the hypothesis that this species could be a source of malodorous oral compounds [4]. *S. moorei* has also been increasingly reported as being associated with other various oral diseases, including different periodontal and endodontic diseases [5,6,7,8]. Apart from being a microorganism present within the oral microbiota, *S. moorei* has mostly been described as a member of the intestinal microbiota. It has been suggested, based on a metagenomic analysis of the fecal microbiome—which revealed a significant association of several anaerobes, including *S. moorei*, with colorectal cancer—that this species might be involved in colorectal carcinogenesis [9]. Besides its role in oral infections, *S. moorei* is considered to be an opportunistic pathogen causing mainly bloodstream and surgical wound infections [10]. However, reported cases of *S. moorei* infections are still relatively rare, as evidenced by a recent review on this subject by Barrack et al. [10]. Considering the endogenous origin of *S. moorei* infections, the source of infection is often assumed to be the oral cavity or the intestinal tract. However, this seems not always relevant, suggesting that *S. moorei* may be present in other endogenous microbiota.

The scarcity of data concerning the involvement of *S. moorei* in human infections led us to carry out a retrospective analysis of 27 cases of infection involving *S. moorei* and observed in two geographically distant French university hospital centers. To broaden the knowledge on the endogenous reservoirs of *S. moorei* and the implications of this microorganism for human infections, we also carried out a review of the sequences available in the GenBank database and in metagenomic databases.

## 2. Materials and Methods

### 2.1. Case Analysis

We conducted a retrospective case analysis of infections involving *S. moorei* among patients hospitalized between 2006 and 2021 in two large regional university hospitals located in the East (University Hospital Center of Nancy) and South (University Hospital Center of Nîmes) of France. All patients for whom cultures from any biological sample yielded *S. moorei* were included. The isolates had been identified in both laboratories using 16S rRNA gene sequencing, which provided accurate identification of the species under consideration. Taxonomic affiliation to the species *S. moorei* was performed using Clinical and Laboratory Standards Institute interpretive criteria [11]. Antimicrobial susceptibility testing was performed according to the recommendations of the antibiogram committee of the French Society for Microbiology (CA-SFM)/European Committee for Antibiotic Susceptibility Testing (EUCAST) in force at the time of isolation [12]. For each patient included, demographic (age, sex), clinical (type of infection, medical history, treatment, and outcome), and microbiological data (associated cultured bacteria if any, antimicrobial susceptibility profile) were collected.

The Institutional Review Board of the University Hospital of Nîmes approved the study (IRB number 21.03.03).

### 2.2. Review of S. moorei in the Literature and Databases

A PubMed search with the terms “*Solobacterium*” or “*Solobacterium moorei*” or “*Bulleidia moorei*” was conducted on 9 March 2021. We then reviewed all articles published in the English language and involving humans.

The NCBI (National Center for Biotechnology Information) database was searched on the same date, for the sequences of *S. moorei* using two approaches that were crossed to ascertain representativeness of the selection. On one hand, we reviewed all the sequences available in the nucleotide database (https://www.ncbi.nlm.nih.gov/nucleotide/, accessed on 9 March 2021) with the same search terms, and on the other hand, we performed a BLAST (Basic Local Alignment Search Tool) analysis of the 16S rRNA gene sequence of the *S. moorei* type strain JCM 10645^T^ (GenBank accession number NR_115130) with selection of the sequences with the highest sequence identity. These latter were analyzed with Blast2GO software available at http://www.blast2go.de (accessed on 9 March 2021) [13] and sequences ≥98.65% identical to the sequence of *S. moorei* were selected. This percentage corresponds to the current threshold delineating a species as established by Kim et al. on the basis of Average Nucleotide Index and 16S rRNA gene sequence similarities comparison [14]. Finally, metagenomic databases were searched using the Integrated Microbial Next Generation Sequencing (IGMNGS) platform, a comprehensive open source tool for processing 16S rRNA microbial profiles for ecology and diversity studies (https://www.imngs.org/, accessed on 16 March 2021) [15]. The 16S rRNA gene sequence of the *S. moorei* type strain was used at the 99% similarity threshold level proposed to represent the species for the search. To determine the host or type of metagenome range of colonization without consideration of relative sequence abundances, the “report.0.tab” file was used (report of positive samples per sample category, e.g., human gut metagenome, human lung metagenome, pig gut metagenome, etc., for which a single hit is sufficient for a sample to be considered as positive). To determine the levels of relative abundance, the reports “report.0.1.tab” excluding rare (>0.1% threshold) and “report.0.01.tab” including only abundant (>1%) sequences were, respectively, used. These three exported spreadsheet files with the number of samples that were positive for the presence of the queried taxonomy for each sample category were manually curated by the removal of all samples that did not include “metagenome” in their designation and of those with no or ambiguous host information.

## 3. Results and Discussion

### 3.1. S. moorei Isolation Characteristics and Antimicrobial Susceptibility

From 2006 to 2021, a total of 27 *S. moorei* isolates had been detected in clinical specimens obtained from 27 patients in both centers. Isolates were Gram-positive, obligatorily anaerobic, non-sporulated bacilli that grew on *Brucella* blood agar supplemented with hemin and vitamin K1 after 2–5 days of incubation. Anaerobic Gram-positive bacilli are a heterogeneous group of bacteria, difficult to identify in clinical microbiology laboratories before the era of mass spectrometry. The development of MALDI-TOF technologies has increased the performance in identifying anaerobes but may still be unsuccessful at identifying certain species. Indeed, major differences still exist between databases of currently available mass spectrometry systems, particularly regarding anaerobes. *S. moorei* is one example of this, being included in the Bruker MS system database (Bruker) [16], whereas it is absent from that of the VITEK^®^ MS system (bioMérieux) used in our two laboratories. Consequently, all 27 isolates in this study were identified by 16S rRNA gene sequencing, revealing that all of them belonged to the species *S. moorei* (>99% of sequence identity only with that of *S. moorei* type strain). *S. moorei* was identified in 17 male and 10 female patients, with a mean age of 59.5 years (range: 25–93 years). This species was mainly identified in mixed aerobic–anaerobic cultures, as observed for numerous cases of infections involving anaerobes (Table 1). Indeed, these infections are mostly endogenous and favored by damaged mucosal/cutaneous barriers, which allow the penetration of anaerobic species into normally sterile tissues, often together with aerobic/facultative anaerobic bacteria [17]. This results in infections involving a more or less complex association of various bacterial species, in which deciphering the pathogenic role of each species remains challenging. Isolation of *S. moorei* as the sole bacterium within a human clinical sample was rarer, representing seven cases in our study, with 6 of the 11 cases of bacteremia (54.5%) and one subcutaneous infection of the ear representing 6.25% of the infections other than bacteremia in our study.

**Figure 1:**
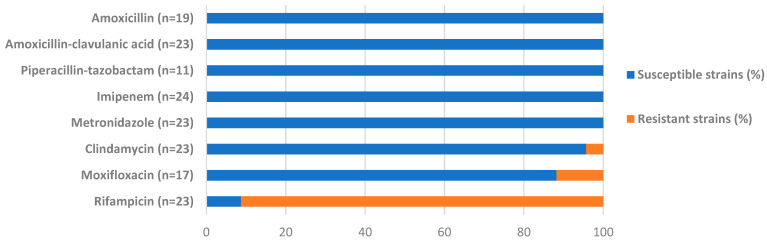
Antimicrobial susceptibility of *Solobacterium moorei* isolates. The antibiotic molecule is indicated on the left side of the figure, followed by, in brackets, the number of strains with available susceptibility data. * Data for ofloxacin were not included in Figure 1 as they were available for 8 *S. moorei* strains only: 3 susceptible strains (37.5%), 3 resistant strains (37.5%), and 2 strains with intermediate susceptibility (25%).

Antibiotic susceptibility data were available for 24 of the 27 *S. moorei* isolates (Figure 1). Resistance to β-lactams and metronidazole was not observed. Resistance to clindamycin (1 of the 23 isolates tested, 4.3%) and to moxifloxacin (2 of the 17 isolates tested, 11.8%) was rarely observed, while 21 of the 23 isolates tested (91.3%) were resistant to rifampicin. With regard to beta-lactams and metronidazole, our data are in agreement with those found in the literature [18,19,20,21,22]. Resistance of *S. moorei* to fluoroquinolones or clindamycin has not yet been reported, whereas resistance to rifampicin has only been reported by Haraszthy et al. in 2008 for oral isolates [4]. It is noteworthy that isolates that were resistant to moxifloxacin, as well as to rifampicin, were responsible for osteoarticular infections for which fluoroquinolones and rifampicin can be used for treatment.

### 3.2. Analysis of S. moorei Infection Cases Revealed a Larger Spectrum of Human Infections Than Currently Described

*S. moorei* was identified during various infectious processes encompassing bacteremia (*n* = 11), skin and soft tissue (*n* = 7), osteoarticular (*n* = 6), central nervous system (*n* = 2), and intra-abdominal (*n* = 1) infections. All of them are presented hereafter successively in comparison with the current literature, with a more detailed description (medical history and treatment) of extremely rare cases or those not described before. Regarding cases not detailed hereafter, most patients had a complex medical history and their clinical conditions required medical and surgical therapy. Medical management most often included broad-spectrum empirical antibiotic treatments subsequently adjusted to the different species identified and their antimicrobial susceptibility profiles. These treatments were mostly active on *S. moorei* regarding the overall susceptibility of the species.

#### 3.2.1. Bacteremia

Bloodstream infection is one of the most frequently reported infections due to *S. moorei*, with 14 cases described so far [10,18,19,22,23]. In our series, *S. moorei* was identified during bacteremia in 11 of the 27 reported cases. This relatively high occurrence might, at least in part, be related to the fact that special consideration is given to isolates obtained from blood in contrast to those that are part of a polymicrobial anaerobic culture obtained from other clinical samples. The first cases of bacteremia due to *S. moorei* were reported in 2006 in patients with multiple myeloma and acute proctitis complicating radiotherapy for cervical carcinoma, respectively [18,19]. These two cases underlined the potential diversity of sources for invasive infections due to *S. moorei*. Indeed, an oral source was suspected based on multiple dentoalveolar abscesses presented by the first patient, whereas, for the second patient, the authors incriminated translocation through the inflamed intestinal mucosa based on the patient’s clinical signs and the initial description of *S. moorei* in human feces. Since then, bacteremia has been reported in 12 additional patients, 10 of whom were recently reviewed [10,22,23]. Common characteristics were that patients had underlying diseases or risk factors (colon cancer, abdominal surgery, diabetes mellitus, etc.) and the most commonly incriminated portals of entry were oral (poor oral condition, dentoalveolar abscess, etc.) or digestive, despite remaining unknown in some cases as not related to any oral or digestive comorbidity [10,22,23]. In our study, an extensive chart review could only be performed for eight patients. For these patients, the portal of entry was likely to be the oral tract (*n* = 1), the gastrointestinal tract (*n* = 3), or the skin (*n* = 2), while remaining unknown for two patients. Comorbidities such as diabetes and/or digestive cancer were also identified in five patients (Table 1).

#### 3.2.2. Skin and Soft Tissue Infections

Skin and soft tissue infections (SSTIs) involving *S. moorei* have been reported in two previous case series [20,22]. Zheng et al. reported nine cases of various SSTIs, including two thigh abscesses, an abdominal wound abscess, an axillary furuncle, two perirectal abscesses, and two pilonidal infections. Although the intestinal origin of the bacterium appeared obvious in some cases, the source of infection remained unknown for others, as in the two cases of thigh abscess and the axilla case furuncle. Whether the bacterium was part of the skin microbiome is still unknown and a potential cutaneous source of infection was not evaluated or discussed. The second study identified *S. moorei* in various samples related to SSTIs of oral origin: a pus sample from hidradenitis suppurativa of the gluteal region, a purulent discharge from the middle ear, an abscess in the mandibular region, and a total laryngectomy wound [22]. For all these cases, *S. moorei* was identified as a member of polymicrobial cultures. Two of the cases reported in our study (Table 1) are remarkable compared with the currently available literature: (i) a case of monomicrobial subcutaneous ear collection in a 22-year-old woman presenting a highly painful abscess of the helix, cartilage damage, and ear pavilion chondritis (case no. 14 in Table 1) and (ii) a breast abscess in a 50-year-old woman with no underlying disease or risk factors (case no. 15 in Table 1).

#### 3.2.3. Osteoarticular Infections

In our study, six patients with bone infection involving *S. moorei* were found (Table 1). The diagnosis of bone infection was made on the basis of clinical, radiological, and microbiological evidence. For all of these patients, *S. moorei* was identified from samples (bone biopsies, *n* = 5; deep collection, *n* = 1) collected under aseptic surgical conditions. For five of these patients, three of whom had diabetes, these infections resulted mainly from the extension of adjacent SSTI, while, for the remaining patient, who presented a mandibular bone infection, the portal of entry was either the oral cavity or the skin. In this case, infection was favored by sequelae (mucosal lesions, cutaneous fistula, and osteonecrosis) of breast cancer treatment (case no. 19 in Table 1).

In the literature, only two case reports of *S. moorei* isolation associated with a bone infection were found: (i) a case of chronic fistulized osteomyelitis of the tibia for which *S. moorei* was isolated among a polymicrobial aerobic–anaerobic culture from serous fluid obtained from the fistula, and (ii) a case of a submandibular abscess for which *S. moorei* was cultured in association with *Fusobacterium necrophorum* from the abscess pus in a patient with suspected chronic osteomyelitis based on CT scan images [22]. However, in both cases, the implication of *S. moorei* in the osteomyelitis remained uncertain regarding the sampling conditions and the absence of bone biopsy analysis [22].

#### 3.2.4. Central Nervous System Infections

To the best of our knowledge, the involvement of *S. moorei* in central nervous system (CNS) infections has so far not been reported, despite the common oral source of these infections and the presence of *S. moorei* in the oral cavity during health and diseases. In our case series, two cases of intracranial abscesses caused in part by *S. moorei* have been observed and have been detailed hereafter, as they represent the first description of the involvement of this species in CNS infections (Table 1).

The first case occurred in a 39-year-old man with a chronic breach of the posterior wall of the frontal sinus resulting from a previous ballistic trauma which had received prophylactic treatment including ofloxacin and rifampicin (case no. 25 in Table 1). The patient presented headaches, epileptic seizures, and sepsis. The computed tomography (CT) scan revealed a collection in the frontal area. Microbiological analysis of the abscess pus showed a mixed anaerobic culture, including an *S. moorei* isolate which was resistant to ofloxacin and rifampicin. The antimicrobial therapy was switched to intravenous cefotaxime, clindamycin, and metronidazole, with a favorable evolution after revision surgery.

*S. moorei* was also identified in a brain tissue sample obtained from a 72-year-old man with headaches, epileptic seizures, and sepsis (case no. 26 in Table 1). The patient had a history of meningioma exeresis with a persistent bone flap infection after craniotomy. CT scanning and magnetic resonance imaging revealed both an intracranial epidural abscess and a frontal brain abscess. Patient management included surgery and meropenem due to associated bacteria, including an extended-spectrum β-lactamase-producing *Escherichia coli*. A favorable outcome was noted for this patient.

#### 3.2.5. Intra-Abdominal Infections

Intra-abdominal infections may represent a possible source of bacteremia, as may be the case for Lee et al.’s case of *S. moorei* bacteremia associated with acute cholangitis [23]. However, it is noteworthy that, until today, no case of intra-abdominal infection in which *S. moorei* has been isolated from intra-abdominal samples has been published. Here, we report a case in which *S. moorei* was directly identified from a necrotic pancreatic collection in a 47-year-old male patient with severe, acute, lithiasic, necrotizing pancreatitis associated with a metabolic disorder (hypertriglyceridemia) (case no. 27 in Table 1). *S. moorei* was co-isolated with *Streptococcus agalactiae*, *Streptococcus anginosus*, and *Staphylococcus aureus*; the patient died despite being treated with piperacillin-tazobactam in addition to surgical debridement (Table 1).

### 3.3. Exploring the Genetic and Metagenomic Sequence Databases for a Complete Spectrum of S. moorei Ecology

#### 3.3.1. Lessons from the Analysis of the GenBank Database

Because sequences deposited in the GenBank database may or may not be associated with published studies, and because sequences originating from metagenomic studies may be associated with published studies not retrieved by using the bacterial name as a keyword for the search unless associated with the main results of the study, we reviewed the sequences present in the database with the aim of obtaining sufficient information about sources of recovery for *S. moorei*. On 9 March 2021, 938 entries corresponded to the search for “*Solobacterium moorei*” and 1151 to the search for “*Solobacterium*” in the NCBI nucleotide database. Most entries corresponded to Whole Genome Sequences, either as scaffold or contig sequences—for example, for strain F024 from the human oral microbiome [24] and strain DSM 22971^T^ (=JCM10645^T^ = RCA59-74^T^), the type strain of *S. moorei* isolated from human feces [1]. After excluding these entries, a comparison with the BLAST search results allowed us to check the completeness of our search and also to exclude sequences which had been erroneously affiliated to *Solobacterium* sp. despite being distantly related to this genus.

**Figure 2:**
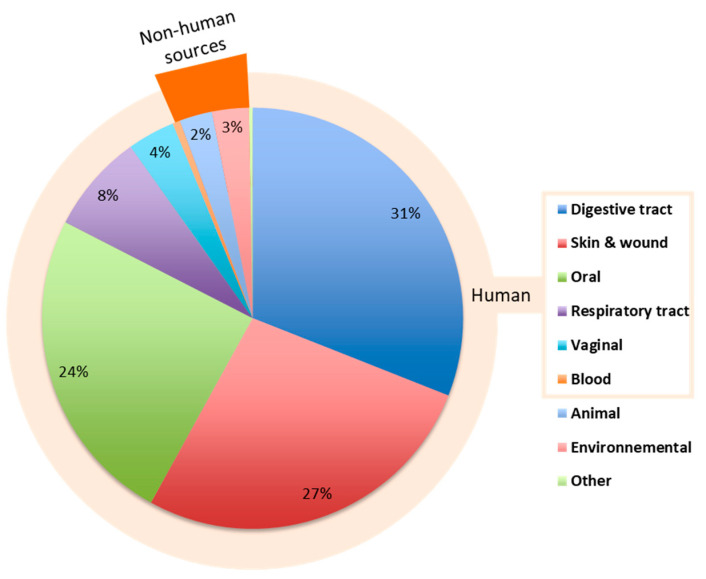
Relative distribution of 16S rRNA gene sequences corresponding to *S. moorei* (≥98.65% of sequence identity with the type strain) in the NCBI database (*n* = 356) according to origin. Sequences in the “Blood” (*n* = 2) and “Other” (*n* = 2) categories each represent 0.56% of the 356 sequences.

A total of 356 sequences sizing from 155 to 1499 nt and corresponding to strains or uncultured clones affiliated to *S. moorei* on the basis of sequences displaying ≥98.65% to 100% identity with that of the type strain were analyzed (Supplementary Table S1). An analysis of isolation sources is presented in Figure 2 and shows that 94% of the sequences originated from human sources (*n* = 336), suggesting that the species is human-associated.

- Digestive tract-related sequences

Sequences related to the digestive tract represented 33% (*n* = 110) of the human sequences and originated either from the esophagus (*n* = 16) or gut/feces (*n* = 94). Human gut sequences were identified from stool and mucosal biopsy specimens (duodenal, colon, cecum) in both healthy [25] and diseased subjects with inflammatory bowel diseases, including Crohn’s disease [26,27,28,29] (Supplementary Table S1). Sequences were identified in adults and children and one sequence corresponded to an uncultured *Solobacterium* sp. clone (clone OTU124/accession number KJ527533) from the stools of preterm infants with extremely low birthweights. The analysis suggested an early implantation of the species in the human gut.

- Oral cavity-related sequences

Around a quarter of the human sequences originated from the oral cavity (26%, *n* = 87) (saliva, subgingival plaque), being part of the human oral microbiome in healthy subjects [24,30,31] but also identified in the presence of tooth decay, gingivitis, and periodontitis, including aggressive forms in line with the known implication of *S. moorei* in oral pathologies [32] (Supplementary Table S1).

- Respiratory tract-related sequences

Probably in relation to the aforementioned presence in the oral cavity, *S. moorei* sequences were also identified in the respiratory tract (*n* = 27). This included uncultured clones from bronchoalveolar lavage fluid, human lungs, the biofilm of extubated endotracheal tube of ICU patients [33], sputum from cystic fibrosis (CF) patients (*S. moorei* strain C1107/JF803577) [34], sputum samples of patients suffering from hospital-acquired lower respiratory tract infection (*Solobacterium* sp. uncultured clone V3 DCM-SHRJH/GU737675), and pneumonia patients with pulmonary emphysema (uncultured bacterium clones KY51_PHKY51_B01_003 and KY51_PHKY51_D05_007 with accession numbers LC260797.1 and LC260830.1, respectively) (Supplementary Table S1). Nevertheless, a pathogenic role of *S. moorei* in respiratory infection is not currently suspected, although its presence in pathologies well-known for their polymicrobial nature involving pathogenic communities such as CF questions the role of this species in the associated dysbiotic community.

- Vaginal tract-related sequences

*S. moorei* was also identified in the vaginal microbiome (*Solobacterium* sp. S4-A19/JX104033) including the vaginal microbiota of HIV-infected African women (13 uncultured clones) [35], as well as in vaginal fluid from subjects with bacterial vaginosis (*S. moorei* strain DNF00973/KU726691) (Supplementary Table S1).

- Skin-related sequences

Likewise, uncultured clones of *S. moorei* were identified from skin at various locations, including the occiput, antecubital and popliteal fossa, volar forearm, external auditory canal, and nostrils [36,37], as well as from human head and neck tissue samples [38], suggesting that the species may also be part of the human skin microbiota (Supplementary Table S1). Finally, six sequences corresponded to strains involved in wound infections described by Zheng et al. [20].

- Blood-related sequences

Only two sequences corresponded to *S. moorei* strains isolated from blood [18,39] (Supplementary Table S1), confirming that not all sequences are deposited in databases, even for published case reports including 16SrRNA gene sequencing for strain identification.

- Non-human-related sequences

Besides human-associated sequences, only nine sequences corresponded to uncultured clones that may be more or less distantly related to other mammals, including the following origins: milk from cows with mastitis, mouse skin [40], a dairy pasteurizer, and an anaerobic digestion reactor (grey cells in Supplementary Table S1). Other unusual miscellaneous sequences were found in the hemolymph of *Crassostrea gigas* oysters, ticks, and in fermenting enset (*Ensete ventricosum*), a kind of banana (orange cells in Supplementary Table S1).

Finally, 10 entries corresponded to uncultured *S. moorei* clones from environmental samples associated with the following sources: soil, soil or post-volcanic pyroclastic surface, volcanic ash at Eyjafjallajökull (Island) deposited in 2010, coal seam environments, Holocene marine sediment, river and river biofilms, mangrove leaves, a jet propulsion laboratory (clean rooms where spacecraft are assembled), and indoor air (green cells in Supplementary Table S1).

Although our search strategy was limited by the information included in the deposit form associated with the sequence, for which the patient’s designation and clinical status are not systematically specified or cannot be systematically found when published (particularly for large metagenomic studies with huge datasets), it brought new insight into the diversity of sources in which *S. moorei* can be identified. The analysis particularly revealed its presence in human vaginal and skin samples and showed us that the species was mainly human-associated.

#### 3.3.2. Lessons from the Metagenomic Database Screening

The presence of 16S rRNA gene sequences displaying close identity to that of the *S. moorei* type strain was found in 55 types of metagenomes for which associated information was available, albeit imprecise for some of them. The analysis was split into human-associated metagenomes (based on “human” or “Homo sapiens” in the designation of the metagenome) and non-human-associated metagenomes. The presence of *S. moorei*-related sequences was overwhelmingly observed in human-associated metagenomes, confirming observations drawn from the analysis of individual NCBI sequences.

**Figure 3:**
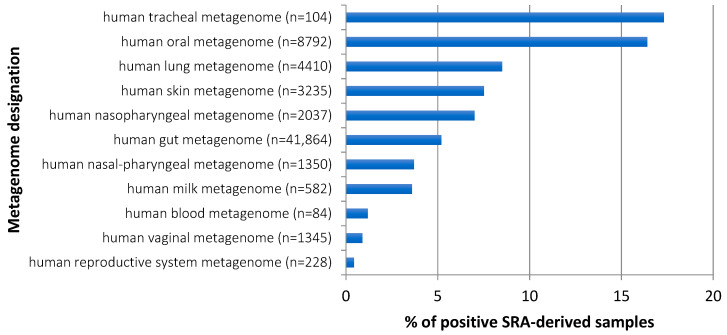
*S. moorei* detection in different human metagenomes.

Regarding anatomical sites, metagenomes that most often contained *S. moorei* sequences were of tracheal and oral origin, with 17.3% and 16.4% of positive samples, respectively (Figure 3). Together with *S. moorei* detection in nasal/pharyngeal metagenomes, this makes the upper digestive and respiratory tracts by far the sites most commonly inhabited by *S. moorei*. By comparison, the presence of *S. moorei* is at least three times less frequently observed in gut metagenomes (5.2% of positive samples) whereas skin metagenomes more often hosted *S. moorei* (7.5% of positive samples) than gut metagenomes. Finally, vaginal and human reproductive system metagenomes also contain *S. moorei* but more rarely than other human metagenomes.

Altogether, *S. moorei* was found to be present in a wider panel of human metagenomes than initially thought from its original description in feces samples and well-known presence in the oral cavity.

**Figure 4:**
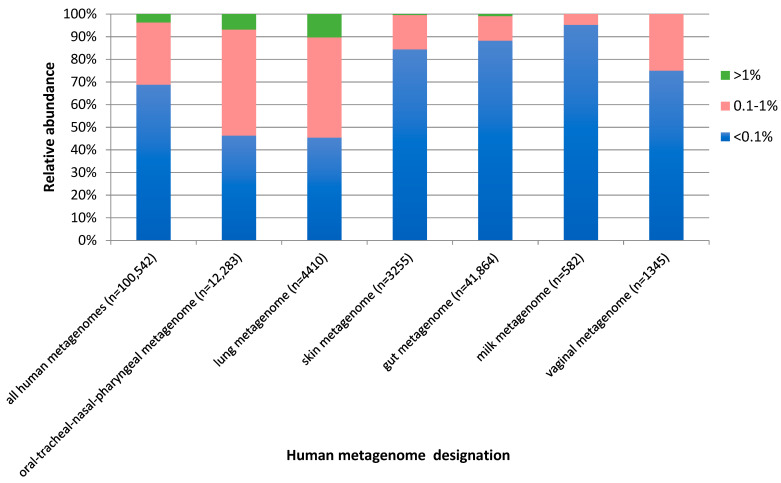
Relative abundance of *S. moorei* sequences in human metagenomes. The type of human metagenome is indicated on the left side of the figure, followed by, in brackets, the number of samples considered in the analysis for each type of metagenome. SRA: sequence read archive.

Regarding the relative abundance of *S. moorei* sequences in human metagenomes, *S. moorei* hits mostly represented rare sequences below 0.1% of the total sequences in 68.8% of positive samples. An abundance of over 1% of total sequences was observed in 3.7% of samples and an intermediate abundance (0.1–1%) in 27.5% of samples (Figure 4). However, variability was observed according to the metagenome considered, with an overall higher abundance of *S. moorei* sequences in samples from the upper digestive and respiratory tracts and the lung (Figure 4).

The type of human metagenome is indicated, followed by, in brackets, the number of sequence read archive (SRA)-derived samples considered in the analysis. Color coding indicates the relative abundance of *S. moorei*-related sequences.

These different observations may support the frequent identification of an oral source or the implication of a digestive source for infection in patients with infectious processes involving *S. moorei*, as detailed in Section 3.1, but also confirm that other human microbiota may also be suspected in certain cases unrelated to any oral or digestive comorbidity. Skin and the reproductive tract are shown to represent alternate portals of entry for *S. moorei* that may have been considered in previous cases of *S. moorei* infection in patients with intravenous drug abuse [21,41] or cervical cancer [19]. In our study, *S. moorei* may have originated from the skin microbiota in at least two cases (subcutaneous ear collection and bacteremia in a 52-year-old patient with diabetes and a recent toe amputation).

In contrast to human metagenomes, *S. moorei* was occasionally identified in various non-human metagenomes, of either animal, environmental, or other origin (Table 2).

This low representation of *S. moorei*-related sequences in non-human metagenomes ties in with the rare, non-human sequences demonstrated in the first part of sequence database analysis of this study and previously shown in Figure 2 with a roughly similar, non-human source distribution (animal, environment, others).

However, although all previous results suggested that *S. moorei* was a human-associated species, atypical observations were made during the metagenome database analysis for pig gut and invertebrate gut metagenomes. These results were therefore more thoroughly analyzed, showing that they should be considered with caution as they probably result from the following biases: invertebrate gut metagenome data originated from very few samples (*n* = 32) resulting from a single study of gut metagenomes in the leech, *Hirudo nipponica*, revealing three *S. moorei*-positive samples. Similarly, 322 of the 325 positive samples from the pig gut metagenome (sequence read archive accession numbers comprised between ERR2739626 and ERR2740414) originated from a single study performing a longitudinal assessment of the gut microbiome in piglets from birth up to weaning. Considering that positive samples originated from single studies and, probably, from non-independent samples in both cases, these potentially biased results are not considered further and *S. moorei* is still considered a human-associated species.

## 4. Conclusions

Taking *S. moorei* as an example, lessons from the GenBank and metagenome databases’ screening are in favor of their usefulness for complementing commonly performed reviews of the literature. Indeed, published data only poorly reflect the growing amount of data available from cultivation-independent studies and sequences deposited in databases. In addition, it is highly probable that most microbiologists did not report the *S. moorei* strains and corresponding cases identified in their institutions as our ‘only’ bicentric, retrospective study doubled the number of cases currently reported in the literature. Our combined strategy, analyzing data from the routine practice of medical microbiology and different databases, allows us to increase the current knowledge on *S. moorei*, an anaerobic opportunistic pathogen in humans. We showed the species to be human-associated and widely present—but not a major representative—in a wider diversity of human metagenomes than previously thought. While mostly present in the oral, lung, and gut microbiota, its distribution among the human microbiota also includes the vaginal and skin microbiota, which may be other sources of infection. As it comes from various microbiota, *S. moorei* is mainly involved in polymicrobial infections where its pathogenic role often remains to be established and where the species is thought to interact with other bacteria. However, these interactions have yet to be characterized. Similarly, the role of *S. moorei* in dysbiotic microbiota associated with gut or lung diseases has yet to be clarified.

## Figures and Tables

**Table 1:** Data for the 27 *Solobacterium moorei* cases with available data included in this study.

Case	Type of Infection	Age (Sex)	Medical History	Specimen	Co-Isolated Species
	Bacteremia				
1		76(M)	Diabetes, sepsis, and peritonitis after hemicolectomy for colon cancer	Blood culture	*Bacteroides thetaiotaomicron, Escherichia coli*
2		85(M)	Not available	Blood culture	*Streptococcus constellatus*
3		65(M)	Diabetes, diabetic foot infection	Blood culture	*Gemella* sp.
4		52(M)	Diabetes, diabetic foot infection	Blood culture	*Peptoniphilus asaccharolyticus* (in other vials), *Staphylococcus aureus*
5		37(M)	Peritonsillar phlegmon	Blood culture	*Prevotella* sp.
6		93(M)	Severe sepsis and diarrhea	Blood culture	None
7		42(F)	Decompensated chronic alcoholic cirrhosis with ascites	Blood culture	None
8		88(F)	Not available	Blood culture	None
9		70(M)	Not available	Blood culture	None
10		36(M)	Not available	Blood culture	Not available
11		75 (F)	Bowel obstruction and appendicular peritonitis	Blood culture	None
	Skin and soft tissue infection				
12	Deep neck abscess	53(M)	Oropharyngeal cancer	Pus	*Streptoccus anginosus*, polymicrobial anaerobic culture
13	Paratracheal abscess	69(M)	Diabetes, lung cancer with thyroid metastasis	Pus	*Anaerococcus murdochii, Eikenella corrodens*, *Staphylococcus epidermidis*, *S. constellatus*
14	Auricular chondritis and abscess	22(F)	Preauricular fistula	Pus	None
15	Breast abscess	50(F)	None	Pus	*Fusobacterium nucleatum*, unidentified *Clostridiales*
16	Infected pressure ulcer	35(M)	Traumatic paraplegia	Biopsy	*Bacteroides fragilis, E. coli*, *Klebsiella pneumoniae*, *Pseudomonas aeruginosa*, *S. aureus*, *Streptococcus dysgalactiae*
17	Infected pressure ulcer	60(M)	Traumatic paraplegia	Biopsy	*E. coli*
18	Stump infection	43(F)	Below-knee amputation for tibial tumor	Biopsy	*P. asaccharolyticus*
	Osteo-articular infection				
19	Mandibular bone infection	84(F)	Breast cancer, mandibular bisphosphonate-related osteonecrosis, submandibular cutaneous fistula, oral lesions	Biopsy	*Enterobacter cloacae* complex
20	Sternal osteitis	77(F)	Diabetes, thoracic injuries after radiation for breast cancer	Collection	*Actinomyces radingae, Capnocytophaga gingivalis*, *Klebsiella oxytoca*, *Morganella morganii*
21	Ulnar osteitis	49(F)	Self-injurious behavior, chronic forearm injury	Biopsy	*Peptostreptococcus anaerobius*, *S. aureus*, *S. anginosus*, *Streptococcus mitis/oralis*
22	Diabetic foot osteitis	71(M)	Diabetes	Biopsy	*Streptococcus agalactiae*
23	Pressure ulcer-related pelvic osteomyelitis	60(F)	Diabetes, ischemic paraplegia	Biopsy	*Actinomyces turicensis, B. fragilis, Clostridium ramosum*, *Finegoldia magna, P. aeruginosa, S. aureus*
24	Pressure ulcer-related femoral osteitis	31(M)	Traumatic tetraplegia	Biopsy	*S. aureus, S. mitis*/*oralis*
	Central nervous system infection				
25	Brain abscess	39(M)	Craniofacial trauma	Pus	*Actinomyces odontolyticus*, *F. magna*
26	Intracranial epidural abscess and brain abscess	72(M)	Meningioma resection	Intracranial tissue	*A. odontolyticus*, *Atopobium parvulum, Bacteroides uniformis*, *E. coli*, *S. anginosus*
	Intra-abdominal infection				
27	Necrotizing pancreatitis	47(M)	Obesity, severe hypertriglyceridemia, biliary lithiasis	Collection	*S. aureus, S. agalactiae*, *S. anginosus*

**Table 2:** Non-human metagenomes containing *S. moorei* sequences.

Type of Metagenome	Samples (n)	Positive Samples (n)		
		All Abundances (%)	Excluding Rare Abundance <0.1%	Abundance >1% Only
Host-associated metagenomes				
pig gut metagenome	3556	325 (9.14%)	58	1
pig metagenome	1162	5 (0.04%)	1	-
plant metagenome	12101	13 (0.11%)	1	-
bovine gut metagenome	9908	11 (0.11%)	8	-
bovine metagenome	1101	4 (0.36%)	1	-
fish metagenome	706	7 (0.99%)	2	-
mouse gut metagenome	19703	6 (0.03%)	-	-
mouse skin metagenome	685	6 (0.88%)	1	-
mouse metagenome	1541	3 (0.19%)	-	-
coral metagenome	2795	4 (0.14%)	2	-
insect metagenome	1544	3 (0.19%)	-	-
invertebrate gut metagenome	32	3 (9.38%)	-	-
beetle metagenome	327	2 (0.61%)	-	-
rat gut metagenome	1422	2 (0.14%)	-	-
fungus metagenome	395	2 (0.50%)	-	-
canine metagenome	228	1 (0.44%)	-	-
nematode metagenome	149	1 (0.67%)	-	-
fish gut metagenome	1000	1 (0.10%)	-	-
primate metagenome	222	1 (0.45%)	-	-
Environmental source metagenomes				
dust metagenome	1018	45 (4.42%)	-	-
soil metagenome	67790	21 (0.03%)	1	-
marine metagenome	37438	18 (0.05%)	1	-
freshwater metagenome	14593	15 (0.10%)	-	-
aquatic metagenome	10493	8 (0.80%)	1	-
indoor metagenome	719	8 (1.11%)	4	-
air metagenome	1047	7 (0.67%)	-	-
groundwater metagenome	745	2 (0.27%)	-	-
freshwater sediment metagenome	1494	1 (0.07%)	-	-
hydrothermal vent metagenome	285	1 (0.35%)	-	-
phyllosphere metagenome	1061	1 (0.09%)	-	-
rhizosphere metagenome	14155	1 (0.01%)	-	-
sand metagenome	87	1 (1.15%)	-	-
sludge metagenome	1924	1 (0.05%)	-	-
terrestrial metagenome	887	1 (0.11%)	-	-
Other metagenomes				
wastewater metagenome	2738	59 (2.15%)	-	-
activated sludge metagenome	2846	29 (1.02%)	-	-
bioreactor metagenome	2376	8 (0.34%)	-	-
anaerobic digester metagenome	1379	1 (0.07%)	1	1
bioreactor sludge metagenome	542	1 (0.18%)	-	-
floral nectar metagenome	487	1 (0.20%)	-	-
food production metagenome	376	1 (0.27%)	-	-
food metagenome	2119	1 (0.05%)	-	-

A sample corresponds to an SRA (sequence read archive)-derived sample; a positive sample corresponds to a sample in which at least one sequence corresponding to *S. moorei* (sequence identity threshold: 99%) was detected (all abundances). The numbers of samples with relative abundance of *S. moorei*-related sequences >0.1% and >1% are given according to the type of metagenome. -: no sample in this category. Bold type indicates atypical, biased observations further discussed in the text.

## Data Availability

Not applicable.

## Supplementary Materials

The following are available online at https://www.mdpi.com/article/10.3390/microorganisms9061229/s1, Table S1: Data associated with the 16S rRNA gene sequences (*n* = 356) displaying ≥98.65% identity with that of the type strain of *S. moorei* (accession NR_115130).
